# Supplementary material for: The Improved Remediation Effect of the Combined Use of Earthworms with Bacillus subtilis-Loaded Biochar in Ameliorating Soda Saline–Alkali Soil
Source: Microorganisms. 2025 May 28;13(6):1243. doi: 10.3390/microorganisms13061243 (PMC12195584; doi:10.3390/microorganisms13061243)
Supplement: Supplementary file 1 [file microorganisms-13-01243-s001.zip › microorganisms-3576654-supplementary.pdf]

Supporting information

Coupling Mechanism of Earthworms and *Bacillus subtilis*-Loaded Biochar in Ameliorating Soda Saline-Alkali Soil

Zhichen Liu[1], Yingxin Huang[2], Qibiao Li[3], Luwen Zhang[1], Zhenke Liu[1], Zunhao Zhang[4], Yuxiang Chen[1]*

1. College of Biological and Agricultural Engineering, Jilin University, Changchun 130022, China

2. Northeast Institute of Geography and Agroecology, Chinese Academy of Sciences, Changchun, 130102, China

3. Zhanjiang Experiment Station, Chinese Academy of Tropical Agricultural Science, Zhanjiang 524000, Guangdong, China

4.The Electron Microscopy Center, Jilin University, Changchun 130000, China;

*Corresponding author. College of Biological and Agricultural Engineering, Jilin University, Changchun 130022, China

E-mail: chen@jlu.edu.cn

Contents

Table S1. Mapping software.

Table S2. Initial carbon component content of soil.

Table S3. Initial key enzyme activity in soil.

Figure S1. Phylogenetic tree of BH-8 strain based on 16S rDNA analysis system.

Table S1. Mapping software

Figure	Mapping software
Figure 1	Origin 2019
Figure 2	Origin 2019
Figure 3	Origin 2019
Figure 4	Origin 2019
Figure 5	Majorbio cloud platform
Figure 6	Majorbio cloud platform
Figure 7	Origin 2019
Figure S1	Majorbio cloud platform

Table S2. Initial carbon component content of soil

SOC (g kg^{-1})	DOC (g kg^{-1})	POC (g kg^{-1})	ROC (g kg^{-1})	HA (g kg^{-1})	FA (g kg^{-1})
5.29	178.27	0.37	2.32	1.35	0.34

Table S3. Initial key enzyme activity in soil

β-glucosidase (μg p-nitrophenol g^{-1} h^{-1})	Invertase (mg glucose g^{-1} $(24\ h)^{-1}$)	Urease (mg NH_4^+-N g^{-1} $(24\ h)^{-1}$)
17.61	0.93	0.19

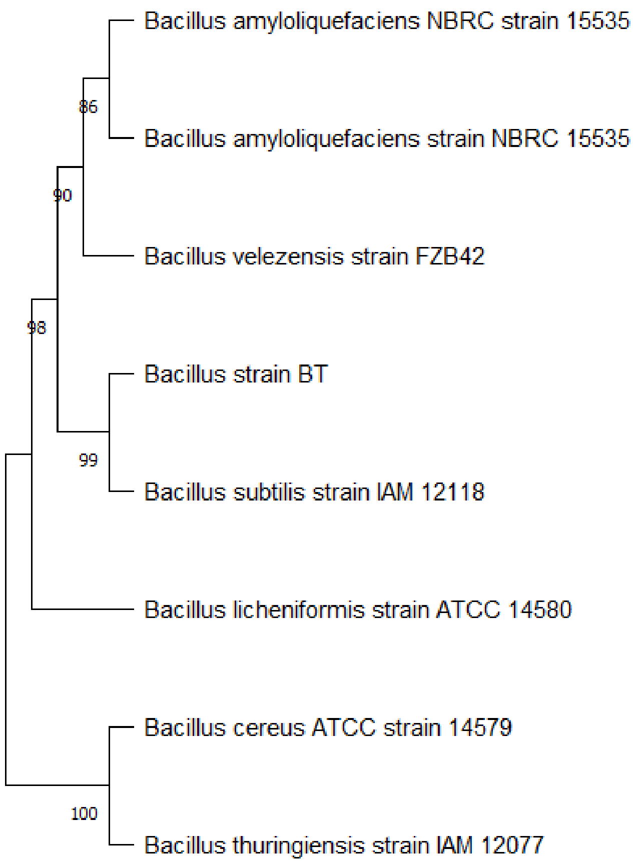

Figure S1 Phylogenetic tree based on 16S rDNA analysis system.
